# Editorial: Stroke and cerebrovascular disease in young adults

**DOI:** 10.3389/fneur.2025.1621692

**Published:** 2025-05-23

**Authors:** Amre Nouh, Ahmed Koriesh, Marc Babi, Whitney Mayberry

**Affiliations:** Cleveland Clinic Florida, Cleveland, OH, United States

**Keywords:** stroke, cerebrovascular disease, intracerebral hemorrhage, young adults, ischemic stroke

## Methamphetamine and stroke in young adults

In this article by Hemphill et al., a case report on methamphetamine use and review of literature illustrates the growing surge in illicit substance use in the US, rising to 1 in 4 adults in 2022, and associated risk of developing stroke. Methamphetamine use has seen increased use and is associated with a higher risk of intracerebral hemorrhage up to 2–5 folds. The related ischemic stroke risks are not as well described as the hemorrhagic counterpart. The proposed mechanisms of stroke are bimodal; acute (hypertensive surge, vasoconstriction and reversible cerebral vasoconstrictive syndrome, BBB breakdown and posterior reversible encephalopathy syndrome and vasculitis) and chronic with small vessel disease and aneurysmal formation and rupture. With the latter, more data is emerging regarding the risk of rupture in patients with occult (or previously known) intracranial cerebral aneurysm and size, morphology and the temporal effects of methamphetamine use. The authors point out diagnostic challenges including false positive urine drug screen with some antipsychotics, antidepressants and antihistamine's that would require secondary confirmatory testing. Furthermore, substantiating chronic users without relevant history would not be feasible using routine screening methods due to metabolite half-life and the many “street” name of the drug may confuse clinicians regarding true use and preparations with contaminants. No proven pharmacological strategies are currently available for methamphetamine cessation, adding some challenge. The authors emphasize the need for adequate recognition, history, screening and early intervention to help with this surging issue and increase stroke risk in young adults.

## Plasma apoA-I and apoB levels and ischemic stroke subtype risks

In this article by Van Tuyen et al., the authors highlight the role of apolipoproteins as in particular apoB may be more useful clinically than LDL cholesterol as it captures greater information about atherogenic particles and is not influenced by heterogeneity of particle cholesterol content and are more studies in cardiovascular disease. In their study of 406 patients with ischemic stroke divided into those with intracranial atherosclerotic disease (ICAS), extracranial atherosclerotic disease (ECAS) and small artery occlusion (SAO), serum apoA-I levels were significantly lower in ICAS compared to ECAS and SAO patients, while apoB levels were higher in ICAS patients. Serum apoA-I levels were significantly lower in ICAS compared to ECAS and SAO patients, while apoB levels were higher in ICAS patients. The authors suggest the findings can contribute in the future to the understanding of the role of these markers in disease prediction and mechanism of stroke in contrast to conventional lipid panels alone. They acknowledge the limitations relating to confoundment, single center nature and lack of details of other influences such as diet and exercise on these markers.

## Taxifolin for neurosurgery-associated early-onset cerebral amyloid angiopathy

Choi et al., describe a young 42-year-old man with a history of childhood traumatic brain injury that required a craniotomy for hematoma evacuation who subsequently presented with recurrent lobar intracerebral hemorrhage (ICH) roughly four decades later, histologically confirmed to be CAA. Taxifolin is a plant flavonoid that has been used as a health supplement for its anti-inflammatory and antioxidant properties with evidence in murine models for treating CAA by inhibiting Aβ fibril formation and promoting Aβ clearance. Serial ^11^C-Pittsburgh compound B positron emission tomography (^11^C-PiB-PET) imaging showed a 24% decrease in global standardized uptake value ratio (SUVR) at 10 months after taxifolin use. The patient reportedly experienced clinical improvement with improved consciousness and reduced recurrent ICH frequency, which the authors suggest may be partly attributable to the potential amyloid-β (Aβ) clearing the effect of taxifolin thought the effect diminished at 15 months. The case raises possibility of human Aβ transmission secondary to neurosurgical intervention resulting in iatrogenic cerebral amyloid angiopathy via seeding. Furthermore, it suggests Taxifolin may have a role in management and PET imaging may be a suitable method for objective quantification of disease burden. The pathophysiology is still not fully understood. Nevertheless, beyond case reporting further investigation is warranted in young adults with CAA-related ICH with history of neurosurgical intervention.

## Testosterone supplementation and stroke in young adults

In this review article by Dengri et al., testosterone supplementation is noted to be on the rise in both recreational and prescriptive forms and the potential implications for stroke risk are reviewed. While limited clinical trials have evaluated stroke risk as a primary outcome, the authors summarize the available literature in this regard pointing that the most robust randomized trial did not find a direct correlation between supplementation and stroke risk. Moreover, current guidelines do not recommend cessation of treatment in the setting of stroke related to use in patients with hypogonadism. The authors highlight four potential mechanisms of ischemic stroke through increased blood viscosity and polycythemia, potentiation of thromboembolism, dysregulation of lipid metabolism and endothelial dysfunction. These factors in patients who may have undiagnosed underlying hypercoagulability, or increased risk for thrombosis due to concomitant cardiovascular disease and paradoxical emboli may potentially be at risk. With a growing use in young adults' and even with older patients without hypogonadism, more light needs to be shed on evaluating its use in patients with cryptogenic strokes and recommendations for secondary prevention, and longitudinal use.

## Computational analysis for the prognosis of carotid dissection

In the study by Bonura et al., the investigators performed a fluid-structure interaction (FSI) analysis of the pathophysiology and evolution of internal carotid artery dissection. In their model, they incorporated patient-specific imaging data and biomechanical modeling to simulate the hemodynamics, intraluminal pressures and wall shear parameters that contribute to dissection propagation and stroke risk. They found that the pressure gradient between the true and false lumen is the main factor in the functional occlusion of the artery lumen and development of stroke symptoms. They were also able to use their model to predict the risk of dissection expansion and the risk of thrombosis. The study highlights the possibility of advanced computational modeling as a tool for developing patient-specific prognostic assessments and treatment planning in carotid dissection.

## Angiographic features and risk of progression in children with moyamoya disease

In their 2024 article, Wang et al. examined the angiographic features that could help in identifying the stroke risk in children with asymptomatic moyamoya disease (MMD). The authors of this study reviewed imaging parameters in 438 cerebral hemispheres and focused on variables such as Suzuki's stage, the development of collateral vessels, and the involvement of PCA to determine the likelihood of the patients to have ischemic or hemorrhagic strokes. The results showed that higher Suzuki's stages were associated with ischemic strokes, while hemorrhagic events were associated with advanced age and PCA involvement. However, the angiographic patterns observed in asymptomatic children were more similar to those of the hemorrhagic group than to the ischemic group, which suggests that these patients may be at relatively higher risk of bleeding in the long term. This research therefore highlights the use of angiographic features to assess stroke risk and possible early intervention in pediatric MMD.

## Cannabis use and the occurrence of stroke in the young population

In 2024, Liu et al. in a systematic review and meta-analysis attempted to establish the effects of cannabis use on stroke incidence in the young. The authors reviewed six observational studies involving more than 119 million participants to determine if cannabis use is a cause of stroke. The results showed a positive association between cannabis abuse and stroke with an overall odds ratio (OR) of 1.14 and rising to 1.21 when smoking and alcohol use were controlled for as potential confounders. There was no clear distinction in the risk of stroke between the ischemic and hemorrhagic subtypes, but the studies had highly variable results. However, there was a high level of heterogeneity in the findings of the included studies. The results of the current study show that cannabis abuse may increase the risk of stroke in young people. The authors also advised that more studies should be conducted to establish the specific cannabis dose and use patterns that may contribute to the risk of stroke and stress the importance of early intervention in this vulnerable population.

## A machine learning analysis of sex-specific risk factors for stroke in young adults

In their 2024 study, Jacobs et al. employed machine learning techniques to explore sex-specific predictors of stroke among young adults. The authors reviewed the National Longitudinal Survey of Adolescent to Adult Health, a nationally representative database, and used the Least Absolute Shrinkage and Selection Operator (LASSO) to identify sex-specific stroke risk factors. The researchers found some stroke predictors that were common to men and women including kidney disease, diabetes, and post-traumatic stress disorder (PTSD). Some risk factors were more associated with men than women, for instance, anxiety and heart disease, while chronic migraine and depression were more associated with women. The differences in the non-clinical predictors were also visible between men and women; men reported alcohol consumption, employment and income whereas women reported education, health access and marijuana use. The study supports the need for personalized prevention strategies and shows that machine learning is useful for stroke risk prediction among young people.

## A nomogram for predicting the risk of cerebral vasospasm after neurosurgical clipping in patients with aneurysmal subarachnoid hemorrhage

Zhou et al. developed a nomogram predictive of cerebral vasospasm (CVS) following clipping following aneurysmal subarachnoid hemorrhage (aSAH) in their 2024 study. The devastating effects of CVS have been recognized for many years; however, predicting the patient population at highest risk has been challenging. A multivariate logistic regression analysis demonstrated age, homocysteine levels, white blood cell count, glucose/potassium ratio, aneurysm location, and modified Fisher grade were independent risk factors for CVS (*N* = 156). A nomogram was developed based on regression analysis with good agreement between prediction and observation in the model and a greater discriminatory performance when compared to independent risk factors. Authors postulate the development of predictive techniques may enhance targeted clinical care during the perioperative period with the goal of preventing detrimental effects of CVS.

## Causal association between depression and intracranial aneurysms: a bidirectional two-sample Mendelian randomization study

In 2024, Wu et al. aimed to better understand the relationship between depression and intracranial aneurysms by analyzing data from publicly available genome-wide association studies of depression. A bidirectional two-sample Mendelian randomization analysis was performed to evaluate for causal relationship. Results demonstrated that genetic propensity for depression was positivity associated with each of the tested groups—intracranial aneurysm, subarachnoid hemorrhage, and unruptured intracranial aneurysm (uIA). A reverse Mendelian analysis was additionally conducted which demonstrated causal effect only in relationship to uIA and depression risk. This study provides further linkage between depression and cerebrovascular risk and prompts further evaluation of associated mechanisms and options for risk mitigation.

## Endovascular treatment for young patients with acute large vessel occlusion stroke in china: analysis of the ANGEL-ACT registry

In review of the ANGEL-ACT registry, Han et al. evaluated stroke etiology and clinical outcomes for younger adults (< 50 years old) following acute treatment with endovascular therapy (EVT). Two hundred and sixteen (13%) of patients who underwent EVT fell within the target age group in the registry; this population garners special interest given greater lifetime disability burden. The primary outcome was modified Rankin Score (mRS) at 90 days with additionally secondary and safety outcomes. When compared to the control population (≥50 years), younger patients had lower median NIHSS, baseline blood pressure, and prevalence of cardiovascular risk factors. Alternatively, prior smoking history was more prevalent in the younger population. Younger patients had a larger prevalence of other and unknown stroke etiology, lower rate of cardioembolism and a higher prevalence of intracranial atherosclerosis. Notably, onset to puncture time was shorter in the control group. Despite this finding, adjusted analysis demonstrated more favorable clinical outcomes in the younger patient population. This study brings continued attention to ischemic stroke burden in young adults and further details associated risk factor prevalence in the Chinese population. Although more favorable outcomes were demonstrated for patients < 50 years of age, the associated societal impact remains significant and will likely represent a continued area of investigative interest.

## Optimal target blood pressure for the primary prevention of hemorrhagic stroke: a nationwide observational study

Shim et al. performed an observational study evaluating the risk of intracerebral (ICH) and subarachnoid (SAH) hemorrhage as it related to blood pressure (BP) measurements for healthy individuals in Korea over a ten-year period. The population included patients aged between 20 and 65 years without history of stroke and took into consideration the 2017 American Heart Association (AHA) staging of hypertension. Study results indicated ICH risk factors were more prevalent in men. The adjusted hazard ratio (AHR) for ICH in women increased with stage one hypertension while higher risk of ICH did not occur in men until they reached stage two hypertension. This difference was attributable to a higher incidence of ICH in the reference group (low normal BP) for men. Regarding SAH, the AHR increased starting at stage one hypertension for both men and women. Prior research established a higher incidence of unruptured aneurysms in women which may explain the higher incidence of SAH demonstrated in women during the observational period. Limitations exist surrounding the generalization of this data as the patient population was exclusively Korean. Nonetheless, authors advocate for aggressive management of blood pressure according to recent AHA guidelines to lower risk of hemorrhagic stroke based on this observational trial.

## Prognostic analysis of endovascular mechanical thrombectomy in stroke patients with acute internal carotid artery obstruction based on circle of Willis variation

This study investigated the prognostic implications of anatomical variations in the circle of Willis on outcomes following endovascular mechanical thrombectomy (EVMT) in patients with acute internal carotid artery occlusion (AIICAO) (Qiu et al.). A retrospective analysis of 108 patients classified AIICAO based on circle of Willis variations, thrombus size, and location. The analysis demonstrated significant differences in patient outcomes, including symptomatic intracranial hemorrhage (sICH), successful revascularization (mTICI), and 90-day modified Rankin Score (mRS), related to the specific anatomical variation. The authors concluded that circle of Willis variations significantly affect outcomes post-thrombectomy, emphasizing their potential use in patient selection and prognosis prediction. This study effectively identified an essential clinical aspect—anatomical variations—that influence treatment outcomes. However, the retrospective design and the relatively small cohort size limit generalizability. Prospective studies would validate and potentially refine these prognostic criteria, improving clinical utility in acute stroke management. In summary: Anatomical variations in the circle of Willis are critical prognostic markers in AIICAO treated with EVMT. Early identification of patients with poor collateral circulation may optimize outcomes.

## The significance of postbypass blood flow model inside to side bypass for moyamoya disease in predicting postoperative cerebral hyperperfusion syndrome

This study explored whether specific blood flow models (BFMs), identified intraoperatively during side-to-side (s-s) bypass procedures for Moyamoya Disease (MMD), predicted postoperative cerebral hyperperfusion syndrome (CHS) (Wan et al.). Analyzing 166 hemispheres from 153 patients, the researchers categorized BFMs into two types (BFM I and BFM II). BFM I exhibited higher CHS incidence compared to BFM II, indicating its predictive significance. The authors suggest that identifying BFMs during s-s bypass may reduce postoperative CHS by guiding clinicians toward appropriate perioperative management. This study provided novel insights into preventing CHS post-surgery for MMD by linking specific intraoperative blood flow patterns to clinical outcomes. However, its single-center design and lack of randomized controls somewhat limit its immediate clinical applicability. Further multi-center validation is essential to confirm the study's practical benefits. In summary, BFM classification during surgery could serve as a valuable clinical tool for predicting and preventing CHS in Moyamoya disease. BFM II, especially under s-s bypass, seems to confer a lower incidence of CHS.

## Burden of stroke in adolescents and young adults (aged 15–39 years) in Southeast Asia: a trend analysis from 1990 to 2021

Using data from the Global Burden of Disease (GBD) study, this study analyzed stroke trends [ischemic stroke, intracerebral hemorrhage (ICH), subarachnoid hemorrhage (SAH)] among adolescents and young adults in Southeast Asia (SEA) between 1990 and 2021 (Satapathy et al.). The study found rising incidence of ischemic strokes, particularly among males aged 30–39 years. Conversely, ICH and SAH incidences and mortality significantly declined, particularly among younger groups and females. Disparities linked to socio-economic status were evident, with poorer countries showing higher stroke incidences and mortality rates. The Philippines notably showed a marked increase in ischemic stroke incidence. This extensive study highlights critical epidemiological trends crucial for public health policy and targeted preventive measures in SEA. Its strength lies in its comprehensive data analysis; however, the inherent limitations of secondary data (e.g., potential under-reporting or misclassification) could somewhat affect accuracy. Addressing socio-economic disparities and implementing targeted interventions appear essential steps for regional stroke management. Despite improving trends in hemorrhagic stroke, the growing burden of ischemic stroke among SEA's younger population requires targeted public health strategies to mitigate long-term disability and economic costs.

## Hypercoagulable states in young adults with ischemic stroke in a stroke belt state: a retrospective study?

This retrospective study hypothesized that the high stroke mortality and recurrence in the US Stroke Belt are due to hypercoagulable states being underdiagnosed among young adults (aged 18–55) (Gordon and Durica). In this study, the investigators reviewed 311 ischemic stroke cases and found that hypercoagulable states were significantly underrecognized during inpatient care (25.57%) compared to post-discharge reviews by vascular neurologists (54.22%). It highlights an evident gap in the inpatient diagnosis and suggests increased awareness and diagnosis of hypercoagulable states could improve stroke prevention. This study successfully identified a potentially significant gap in the diagnosis of hypercoagulable states that may affect patient outcomes. While its retrospective nature and single-center approach limit generalization, it strongly suggested a need for broader awareness and standardized diagnostic protocols in young stroke patients, particularly in high-risk regions like the Stroke Belt. Hypercoagulable conditions are likely a major and under detected contributor to stroke in younger adults. Increasing diagnostic vigilance, including systematic testing and follow-up, may lower recurrence and mortality in high-risk regions. In summary, Hypercoagulable conditions are likely a major and under detected contributor to stroke in younger adults. Increasing diagnostic vigilance, including systematic testing and follow-up, may lower recurrence and mortality in high-risk regions.

